# Is the evaluation of performance gender specific? Evidence from two large experimental studies

**DOI:** 10.1371/journal.pone.0336066

**Published:** 2026-02-18

**Authors:** Axel Franzen, Fabienne Wöhner

**Affiliations:** Institute of Sociology, University of Bern, Bern, Switzerland; Xi'an University of Posts and Telecommunications, CHINA

## Abstract

It is often assumed that women receive less recognition than men for the same work or performance. We test this assumption via two large-scale online-survey experiments involving 3,157 (Study I) and 2,909 (Study II) respondents respectively. In both studies, subjects watched a two-minute video in which either a female or a male character presented either a male-associated topic, a gender-neutral topic, or a female-associated topic. Since former research suggests that the attractiveness of the presenter increases performance evaluations, we also varied the attractiveness of the presenting characters. Study I uses a 2 (gender) by 2 (attractiveness) by 3 (topic) design. The video presentations were created using an artificial intelligence video-maker that used human-like avatars. In Study II, the presentations were conducted by real humans. The findings from both studies suggest that gender has no influence on how respondents evaluate presentations. Additionally, in Study I we find that attractive male avatar presenters receive more favorable evaluations than less attractive males. In contrast, female avatar presenters do not receive an attractiveness bonus. However, when using real humans instead of avatars we did not find this attractiveness bonus for men.

## 1. Introduction

There is extensive empirical evidence suggesting a gender bias in performance evaluations. Almost all studies investigating the wage structure of labor markets report a gender wage gap. Many studies, particularly those in Europe and the US, find that the wage gap has declined during the last decade. However, recent studies also report that women receive about 8% less pay than men even after controlling for observed productivity-relevant characteristics such as schooling, experience, occupation and the business sector in which they are active [1–3]. Evidence that women encounter a disadvantage in performance evaluations also comes from studies on student evaluations of teaching [4]. Even though the results from these studies are mixed, most find that female professors receive lower teaching evaluations as compared to their male colleagues [4–9] (but see for instance [10] which reports no gender bias in evaluations).

Labor market studies suffer from the disadvantage that some productivity-relevant characteristics might be unobserved. Hence, wage differences might not be due to gender but due to unobserved productivity-relevant characteristics such as work effort, career ambitions and so on. This disadvantage also applies to analyses of student evaluations. Many universities assign faculty members randomly to classes which use the same course materials. This fact is utilized in some studies investigating gender effects in student evaluations [8]. But even random assignment using the same class materials leaves some room for differences in teaching style or the way professors interact with students during a semester. Hence, it is important to complement existing observational studies with randomized experiments in which everything besides the presenter's gender is held constant. Some new, relevant experimental studies are reviewed in the next section. Most existing experimental studies vary the presenter's gender simply by altering the surname or username attached to a certain task. This is a very subtle stimulus and might not be noticed by every participant. The two studies we present report the results of two online experiments in which the participants (students in Study I, general Swiss population sample in Study II) viewed two-minute video presentations. We randomly assigned one video to each respondent and let them evaluate the presenter and their talk. In the videos, we varied the gender and attractiveness of the presenter, as well as the topic of the presentation. The findings suggest that gender had no influence on how respondents evaluated the presentations.

The remainder of this paper is organized into seven sections. The next section describes the findings of former experimental studies on the gender and attractiveness bias. The section closes with four hypotheses that guide our experimental studies. Section 3 describes the experimental design of Study I. The results of the first experiment using student subjects are presented in section 4. Section 5 discusses the result of Study I and addresses some of its limitations. In section 6, we describe the design of Study II which we conducted in order to address the limitations of Study I. The results of Study II are described in section 7. Finally, the findings are discussed and summarized in section 8.

## 2. Theory and experimental evidence

There are relatively few studies that investigate gender differences of performance evaluations via an experimental design [11]. Many of these studies are conducted online. One example is the field experiment by Bohren et al. (2019) [12]. The authors posed questions and answers in a large online mathematics forum. In addition to posting questions and providing answers, users of the forum can evaluate (upvote or downvote) the quality of other users’ questions and answers. These evaluations result in reputation points for users. Every user of the forum can be identified by a username and his or her reputation score. Bohren et al. [12] generated new accounts (N = 140) with female or male usernames and posted questions and answers to the forum. With respect to posted answers, they did not find gender differences in the reputation scores received for answers. However, accounts with female usernames received lower reputation scores for questions as compared to accounts with male usernames. However, this difference in received reputation for questions held only for novice accounts that started with low or no reputation scores. For usernames with high reputation scores, female accounts received even higher reputation scores than male accounts. The authors interpreted these findings as evidence of statistical discrimination against women. Discrimination is absent if the quality of a post can be assessed objectively. This is the case for answers, since they can be either right or wrong. Quality assessments of questions are much more ambiguous. Under this condition, female usernames receive a lower reputation score. However, if the usernames are accompanied with high reputation scores, female accounts receive even higher reputation scores than male accounts. The authors’ explanation of this latter finding is that users give an extra bonus to female accounts if they were high achievers in the past.

Neschen and Hügelschäfer (2021) [13] presented students (N = 127) with different profiles of other students who had to solve math problems in another experiment. The participants were told that students solved on average 16 math problems within eight minutes. The task of the participants was to estimate the number of correctly solved math problems for every student profile. The profiles contained information about students’ age, gender, and subject of study. The results showed that the estimates for male students were higher (M = 16.36) than for female students (M = 15.68) independently of all other characteristics shown suggesting a gender bias. This result was replicated by the authors in a second study [13].

Binderkrantz et al. (2022) [14] discuss five experimental studies of teaching evaluations conducted in the US, Canada, and Germany. Three of them found higher student evaluations for male teachers as compared to female teachers [15–17], while two found no overall evaluation differences [18,19]. The authors also report the results of two of their own experiments. In one study, 262 students of political science were asked to view a slide presentation that included a voice over. The gender of the presenter was manipulated by assigning either a female or male name that was also shown on the slides. The authors did not find any statistically significant differences in the evaluation of the online presentations for male or female presenters. This result was replicated in a second study in which student participants (N = 355) viewed different slides, but, in contrast to the first study, did not hear a (neutral) voice. Gender was randomly manipulated by assigning different names to the slides.

Taken together, existing experimental studies of a gender bias in performance evaluations show mixed results. Most studies report that women are evaluated less favorably than men. This applies particularly to studies related to mathematical tasks, and to studies conducted in the US. Remarkably, all the reviewed experiments manipulated the gender of the presenter only by applying either a female or male name. Hence, the manipulation of the stimulus is weak and might have failed to introduce gender stereotypes. In order to apply a stronger stimulus, we generated two-minute video clips in which a female or male person presented either a talk on a male-associated topic, a gender-neutral topic, or a female-associated topic. We hypothesize that females are evaluated in general less favorably than males, regardless of the topic of the presentation (H1). However, according to gender-role incongruence theory [20–23] this disadvantage should be particularly visible for male-affine topics and less strong for neutral or female-affine topics (H2).

Furthermore, past experimental research has shown that more attractive individuals have an advantage in the labor market [24], and are evaluated as more competent, intelligent or social than less attractive people [8,25,26]. Moreover, some evidence suggests that this effect is stronger for males than females [27,28]. Accordingly, we hypothesize that attractive males are evaluated more favorably than less attractive males (H3). But for women, attractiveness can be disadvantageous, particularly in managerial positions or masculine job environments [29,30]. Gender stereotypes imply that females are more “communal” and compassionate than men, who are seen as more “agentic” and accomplishment-oriented [22,31]. Since attractive women are often perceived as more feminine [24,32] attractiveness can increase the gender stereotype. Hence, attractive women might have a disadvantage as compared to less attractive females (H4) when it comes to performance evaluations.

## 3. Materials and methods of study I

The first experimental design (Study I) that was used to test the four hypotheses had a 2 (gender) by 2 (attractiveness) by 3 (topic) design, and accordingly consisted of 12 conditions. The experiment was embedded in an online survey conducted in April through August 2022 at the University of Bern in Switzerland. Emails containing the link to the survey were sent through the university's student admissions office to all Bachelor and Master degree students. Participation was voluntary and written consent was obtained. The study received ethical clearance from the IRB of the Faculty of Business, Economics and Social Sciences of the University of Bern (serial number 022022). The main topic of the survey was the motivation and study situation of students at the University of Bern. The video was placed in the middle of the survey, followed by questions asking the participants to evaluate the presenter and the talk. We prepared 12 videos and randomly assigned each participant one video. In order to ensure that participants watched the full two-minute video, it was impossible to fast-forward the presentations. Furthermore, the “continue” button of the questionnaire on this page appeared only after two minutes. We generated the presentations using Synthesia [33], a video-maker that uses artificial intelligence. Synthesia provides various human-like avatars. The avatars are based on pre-recorded real actors. Former research suggests that subjects react to representations of human-like characters similarly to the way they react to real humans [34–37]. This also applies to the application of gender stereotypes [38]. Synthesia converts texts into female- or male-sounding audio tracks. The movements of the avatars’ lips and faces align with the texts. The software can be used in many different languages and the texts are read as submitted. We rehearsed every presentation several times and adapted the speed and interruptions so that they sounded realistic in German. We picked one attractive female avatar, one less attractive female avatar, one attractive male avatar and one less attractive male avatar. The level of attractiveness of the characters chosen was rated by a group of six students beforehand. We also added a natural-looking background from inside the university building so that the videos looked realistic. Images of the four characters are shown in S1 Fig in S1 File in the supporting information.

Each character presented all three topics. One presentation was about solar radiation management systems, a geoengineering topic. Since this concept is rather technical it was chosen to represent a male-affine topic. The second topic was about a training method for competitive ski racing. The technique is called whole-body vibration training and is gender-neutral since it is practiced both in female and male ski racing, which are both very popular in Switzerland. Finally, participants watched a presentation about a therapeutic method called the Marte Meo Technique. Because pedagogical therapy and counseling is predominantly practiced by women, the topic is considered female-affine. All three talks consisted of 251 words and the video presentations lasted two minutes each. Since each technique was probably new to most students, the presentations were designed to describe them briefly, and to mention both their advantages and disadvantages. However, in each presentation the presenter ended the talk by saying that she/he believes that the method's advantages outweigh its disadvantages. The translated version of the wording of each text is displayed in S1 Table in S1 File through S3 Table in S1 File in the supporting information.

Overall, the videos were assigned to 3,157 students. Since we had 12 experimental conditions, the expected number of observations in every condition was 263. The observed distribution did not differ statistically significantly from this expectation with one exception (see S2 Fig in S1 File in the supporting information). The videos were followed by four questions concerning how competent, attractive, likable, and intelligent the respondents rated the presenters. All four questions were accompanied by 11-point answering scales ranging from 0 (not at all) to 10 (very much). Thereafter, the respondents received two questions concerning the evaluation of the presentation. The first question read “How would you grade the presentation?” and was accompanied with the Swiss grading scale running from 1 (very bad) to 6 (excellent). The second question read “How convincing do you find the method presented in the talk?” This question was accompanied by an 11-point answering scale ranging from 0 (not at all convincing) to 10 (very much convincing). Our focus in the study was how the different stimuli manipulated by the experimental design (gender, attractiveness, and topic of the talk) affected the evaluation of the presentations. Overall, 3,027 respondents completed the evaluations of the presentations. Finally, we also collected respondents' gender and age at the end of the questionnaire. The sample consists of 65.5% female students, 33.5% male students, and 1% of students with a gender identity other than female or male. The mean age is 24.5 years. The descriptive statistics of all variables used in this study are presented in S4 Table in S1 File in the supporting information. For the statistical analysis we calculated two-sided t-tests as well as ordinary least square regressions with robust standard errors (OLS). The critical significance level for hypotheses testing is α = 0.05. We used Stata 19 [39] for the data analysis.

## 4. Results of study I

**Fig 1:**
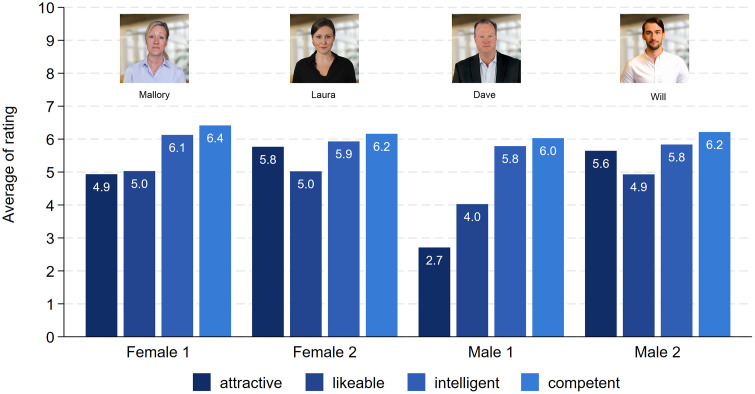
The evaluation of the presenters. Note: Female 1: attractive (N = 789), likeable (N = 795), intelligent (N = 798), competent (N = 799). Female 2: attractive (N = 753), likable (N = 754), intelligent (N = 752), competent (N = 754). Male 1: attractive (N = 788), likeable (N = 787), intelligent (N = 786), competent (N = 785). Male 2: attractive (N = 686), likeable (N = 686), intelligent (N = 685), competent (N = 686).

After viewing the videos, the respondents were first asked to rate, on an 11-point answering scale, how attractive, likable, intelligent, and competent the presenters were. Fig 1 displays the distribution of the attributes assigned to each character. The attractiveness rating differed very much for the two male characters, as intended by the experimental design. Dave, the less attractive male, received a rating of 2.7, as compared to Will, who received an attractiveness score of 5.6 (t = 25.01, p < 0.001). This confirms that the attractiveness manipulation was successful. In principle, this applies also to the two female characters but to a lesser degree. Mallory, the less attractive character received a score of 4.9, and Laura a score of 5.8. This difference is highly statistically significant (t = 7.78, p < 0.001). In terms of likability, intelligence, and competence, there are no relevant differences between the two female characters. This applies also to the two male characters in terms of competence and intelligence. However, participants rated Dave as somewhat less likable (4.0) as compared to the more attractive Will (4.9) (t = 7.47, p < 0.001). More importantly, concerning Hypothesis 1, there were only small differences with respect to the intelligence and competence ratings of the female and male characters. For both attributes, the females had a small advantage of 0.2 points on the 11-point rating scale (t = 2.87, p = 0.004 and t = 2.12, p = 0.034). The same applies to the likability rating, except for Dave, the less attractive male character. This conclusion also holds when we compare the intelligence or competence rating of every character separated by the three presentation topics (results not shown). Hence, the intelligence or competence rating of the characters did not depend on the topic of their presentations.

**Fig 2:**
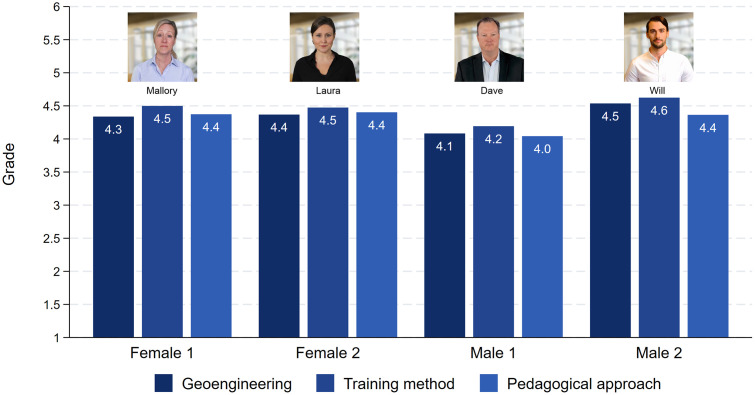
Grades for the presentations. Note: Female 1: Geoengineering (N = 261), Training methods (N = 261), Pedagogical approach (N = 274). Female 2: Geoengineering (N = 264), Training methods (N = 265), Pedagogical approach (N = 225). Male 1: Geoengineering (N = 280), Training methods (N = 257), Pedagogical approach (N = 252). Male 2: Geoengineering (N = 236), Training methods (N = 236), Pedagogical approach (N = 216).

The crucial question with respect to Hypothesis 1 is how the respondents graded the presentations. These results are displayed in Fig 2. As can be seen, all three topics received very similar grades, and the grade did not depend on whether the presenter was female or male. Both Mallory and Laura received average grades between 4.3 and 4.5. Will’s grades were similar to those of the women, ranging from 4.4 to 4.6. Only the grades for the less attractive male were a little lower, ranging from 4.0 to 4.2.

**Fig 3:**
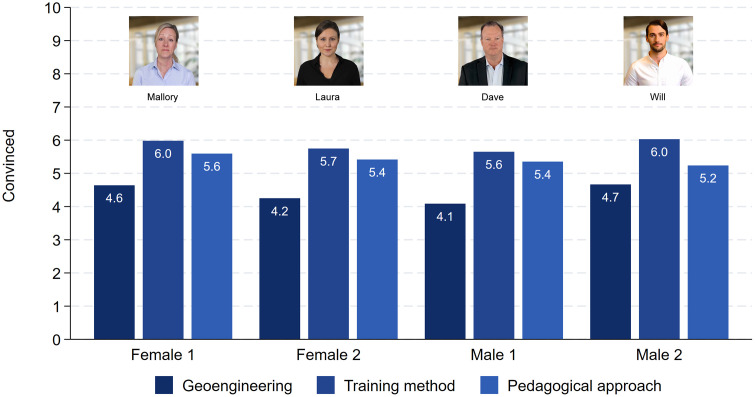
The perceived convincibility of the presentations. Note: Female 1: Geoengineering (N = 263), Training methods (N = 261), Pedagogical approach (N = 273). Female 2: Geoengineering (N = 264), Training methods (N = 265), Pedagogical approach (N = 225). Male 1: Geoengineering (N = 280), Training methods (N = 257), Pedagogical approach (N = 252). Male 2: Geoengineering (N = 236), Training methods (N = 236), Pedagogical approach (N = 216).

The results relating to how convincing the presentations were are presented in Fig 3. The results show that there are no statistically significant gender differences. The two female characters received scores of 4.6 (Mallory) and 4.2 (Laura) for the talk on geoengineering (t = 1.99, p = 0.048). Dave, the less attractive male, received only a score of 4.1 and Will a score of 4.7 (t = 2.75, p = 0.006). Thus, if anything, we see an attractiveness bonus for males but no difference between the female and male characters. Similar results also apply to the other two topics. The score ranges between 5.6 and 6.0 for the ski racing training method, but there are no gender differences. As hypothesized, the two female characters received slightly higher scores for the pedagogical topic (5.6 and 5.4), but the differences compared to the male scores were small (5.4 and 5.2) and statistically not significant. Taken together, neither gender had an advantage or disadvantage that was specific to the topic.

**Fig 4:**
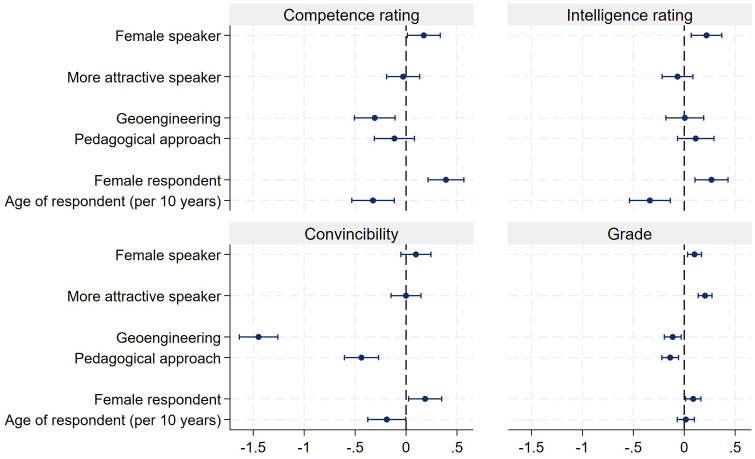
Results of OLS regressions for competence and intelligence ratings, as well as for how convincing presentations were and grade. Note: N of competence rating = 2,987 (adj. R^2^ = 0.014), N of intelligence rating = 2,984 (adj. R^2^ = 0.011), N of convincibility = 2,992 (adj. R^2^ = 0.084)**,** N of grade = 2,991 (adj. R^2^ = 0.018).

The results displayed in Fig 1 to 3 are reconfirmed by analyzing the data via ordinary least squares (OLS) regressions. We use the presenters’ competence and intelligence ratings as well as how convincing they were, and the grades respondents assigned to the presentations, as dependent variables and regress them on the experimentally varied presenter's gender, attractiveness (lower/higher), and subject of presentation. Furthermore, we control for respondents’ gender and age. Because of the low number of observations, we exclude the 29 individuals who did not identify with either male or female gender. Listwise deletion is used in all regression models. The results of the four independent regressions are displayed via coefficient plots [40] in Fig 4 (see also S5 Table in S1 File in the supporting information). As can be seen from the coefficient plot, female presenters did not encounter a disadvantage. Quite to the contrary, females were rated as being more competent and more intelligent as compared to the male presenters. Also, female presenters received slightly better grades than males. All effects are relatively small. For the competence rating, women received 0.17 more points on the 11-point scale than men, a 0.21 better intelligence rating and a 0.10 better grade. These results clearly reject Hypothesis 1. Also, the presenters’ attractiveness had no effect on the competence or intelligence rating. However, attractive presenters did receive a better grade, by 0.21 points, on the grading scale ranging from 1 to 6. Also, the topic of the presentations did have some effect. The presentation about geoengineering was considered least convincing by the respondents and the topic also received somewhat lower grades (−0.11). The plot in Fig 4 also reveals that female respondents were generally more positive in their evaluations. They assigned higher ratings for presenters’ competence and intelligence, as well as for how convincing they were and their grade. Furthermore, older students seem to be more critical. The effect for age is negative for competence, intelligence, and how convincing presentations were.

**Fig 5:**
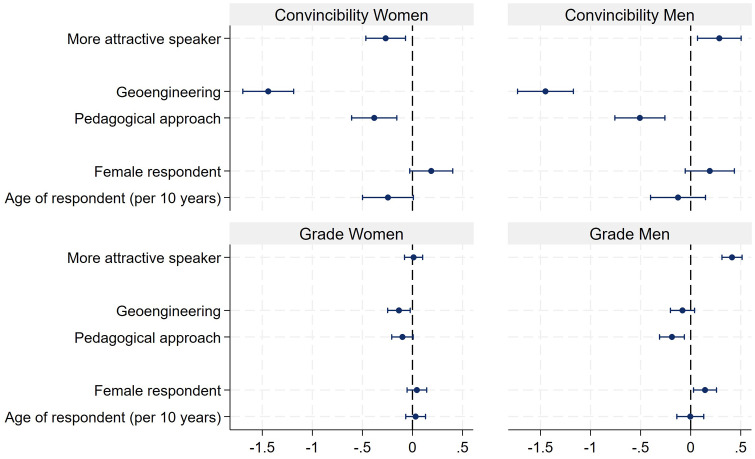
OLS regressions for how convincing presentations were, and the grade of the presentation, separated by the gender of the presenter. Note: N of convincibility for women = 1541 (adj. R^2^ = 0.093), N of convincibility for men = 1,451 (adj. R^2^ = 0.081), N of grade for women = 1,540 (adj. R^2^ = 0.001), N of grade for men = 1,451 (adj. R^2^ = 0.049).

Finally, to investigate the question whether both genders profit equally from attractiveness, we ran separate regressions for female and male presenters on how convincing presentations were, and their grade. The results are displayed in Fig 5 (see also S6 Table in S1 File in the supporting information). As can be seen, the more attractive male presenter (Will) received a higher score with regard to being convincing (0.29) and also received a higher grade (0.41) than the less attractive Dave. This attractiveness bonus was not found for the female presenters. The more attractive Laura received even lower scores with respect to being convincing (−0.26). However, there was no difference in terms of the grade.

We conducted a number of robustness checks for the 4 regression results presented in Fig. 4. First of all, multicollinearity is no problem in the regression analyses (VIF vary between = 1.00 and 1.33). In order to take care of heteroscedasticity, we used robust standard errors for calculating the significance levels of regression coefficients. Furthermore, we checked the age variable for nonlinearity by introducing a squared age term into the regression. The results show that there is no nonlinearity with respect to the age effect. Finally, we also checked if statistical outliers bias the estimation results. Also, this test was negative.

## 5. Discussion of study I

Using a survey experiment, the aim of Study I was to investigate if there is a gender or attractiveness bias in performance evaluations. Taken together, we do not find any disadvantage for female presenters in terms of their competence and intelligence rating. Quite to the contrary, the female presenters received a slight advantage as compared to male presenters. This also held true for the grade respondents assigned to their talks. However, there was no difference with respect to the convincibility of the presentations. Hence, these results clearly refute Hypothesis 1 that female presenters receive less favorable evaluations as compared to male presenters. These results also hold true when we look at different topics. The topic of the talk mattered for the evaluation with respect to how convincing it was, and the grade. The talk on geoengineering always received lower evaluations as compared to the two other topics. However, this result was independent of the gender of the presenter. Hence, this result also refutes Hypothesis 2. According to our results, women face no disadvantage in evaluations, even when they present a technical topic.

Attractiveness turned out to be an advantage for the male characters. This result supports our Hypothesis 3 and aligns with former findings in the literature [8,27]. However, this attractiveness bonus was not observed for women. There was no difference in terms of the grade assigned to the more attractive Laura as compared to the less attractive Mallory. Moreover, the more attractive female character actually received a lower evaluation in terms of how convincing her talk was. Hence, this finding only partly supports (or refutes) Hypothesis 4.

We believe that the experiment we conducted has a number of advantages over past experimental studies. First, we used a large sample, enabling us to run an extensive design with 12 conditions allowing to vary not only gender but also attractiveness and topics. Second, we varied gender not by merely changing the names assigned to slides or presentations but by presenting videos of female and male characters, and hence induced a very visible and noticeable stimulus. Third, using artificial characters allowed for a very high level of standardization between the presentations. In particular, every presentation had the exact same number of words and speed of content presentation. Fourth, the voices differed only between the genders but were the same within the gender variation. This isolated the attractiveness of presenters’ appearance from any other way of presenting the topic.

However, Study I also has some limitations. Most importantly, we conducted the experiment at one university among students. Hence, the sample consists of highly educated young adults and demonstrates that women receive equivalent evaluations as compared to men in an academic context. The results cannot be generalized to the Swiss population. It may very well be the case that gender stereotypes have disappeared in the academic student population but are still in place in the general public. Second, we used artificial characters. This allowed us to keep the presentations very similar, apart from the gender of the presenter. But, at the same time, the characters did not appear as real as real human beings. Hence, there might be the possibility that the avatars were not perceived as humans but as neutral robots to which gender stereotypes do not apply. Thus, the perceived artificiality of the avatars might have concealed the gender effect.

## 6. Materials and methods of study II

To address both arguments, we conducted a second study in March 2024 using a non-student sample and real humans as presenters in addition to avatars. As a sample, we selected 2,909 individuals from the 110,000 participants of one of the largest providers of online-panels in Switzerland (Intervista). Participation was voluntary and written consent was obtained. The study received ethical clearance from the IRB of the Faculty of Business, Economics and Social Sciences of the University of Bern (serial number 092024). Participants were restricted to the German speaking part of the panel. Furthermore, the panel was pre-stratified according to gender and age and participants were then drawn randomly from these groups. To assess the representativity of the resulting sample, we compared the means and proportions of key demographic variables as well as some attitudinal variables with two large-scale random samples (Swiss Household Panel (SHP), and MOSAiCH) which are widely used for official statistical and scientific purposes in Switzerland. The results of comparing the three samples are depicted in S7 Table in S1 File in the supporting information. Comparison of the demographic characteristics (age, gender, education, number of household members) suggests that our sample from Intervista is similar to the other two random samples. There are two exceptions to this observation: the Intervista sample contains a lower share of employed individuals (77% versus 87% and 84%), and has a lower share of married individuals (41% versus 52% in the other two surveys). Furthermore, the personalized household monthly income is somewhat lower as compared to the SHP (5,900 CHF versus 6,749 CHF).

In addition to comparing demographic variables, we also implemented some attitudinal questions taken either from the SHP or the MOSAiCH into the Intervista sample. The results demonstrate that the Intervista sample is relatively close in terms of the political left-right orientation to the SHP. Minor differences can be observed with respect to individuals’ general trust and general life satisfaction. Both averages are a little lower in the Intervista sample. The MOSAiCH sample contained a number of questions related to attitudes about the role of women in society of which we implemented three into the Intervista sample. Also, here the observation is that the averages are relatively similar. Hence, overall, we conclude that the Intervista sample represents the German speaking part of the Swiss population rather well.

Instead of using preprogrammed avatars as presenters, we hired actors who presented the same three texts we used in Study I. We hired two female and two male professional actors for Study II. As in Study I, we varied attractiveness of the female and male characters by rating different candidates beforehand. Moreover, we used pictures of the real actors to construct avatars. Hence, Study II had a 2 (human vs avatars) by 2 (male versus female) by 2 (attractive versus less attractive) by 3 (talk on geoengineering versus training methods versus pedagogical approach) design with 24 experimental conditions. Each subject received randomly one of the treatments resulting in about 120 observations per treatment. In this paper, we focus on the evaluation of the video talks presented by real people (actors), as this overcomes the limitation of Study I. The number of respondents who saw a video with an actor was 1,415. Respondents were asked about the topic of the presentation in an attention check. Twenty-five individuals could not correctly answer the question and were excluded from the analyses. This results in a final sample size of 1,390. The corresponding descriptive statistics for the variables used in Study II are presented in S8 Table in S1 File in the supporting information. For the statistical analysis we calculated two-sided t-tests as well as ordinary least square regressions with robust standard errors (OLS). The critical significance level for hypotheses testing is α = 0.05. We used Stata 19 [39] for data analysis.

## 7. Results of study II

**Fig 6:**
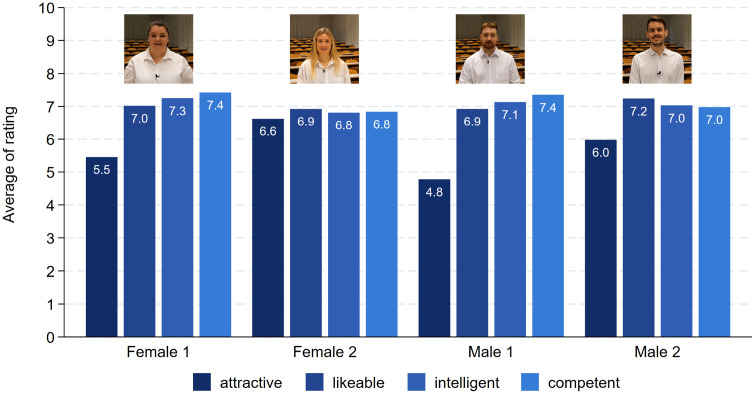
The evaluation of actors. Note: N Female 1 = 335. N Female 2 = 342. N Male 1 = 353. N Male 2 = 360.

**Fig 7:**
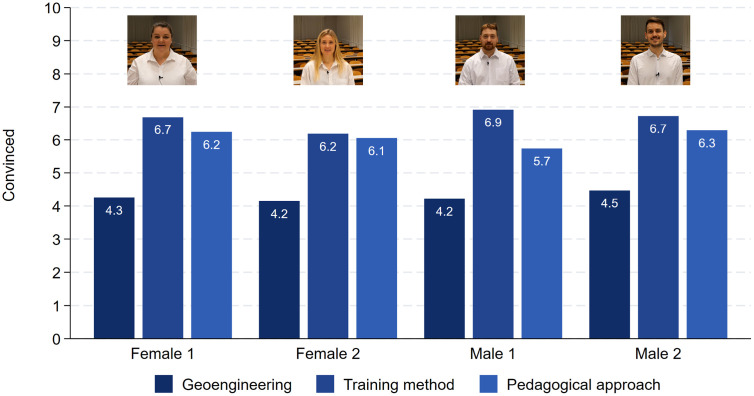
Rating of convincibility. Note: Female 1: Geoengineering (N = 116), Training methods (N = 93), Pedagogical approach (N = 126). Female 2: Geoengineering (N = 120), Training methods (N = 126), Pedagogical approach (N = 96). Male 1: Geoengineering (N = 111), Training methods (N = 108), Pedagogical approach (N = 134). Male 2: Geoengineering (N = 128), Training methods (N = 113), Pedagogical approach (N = 119).

Fig 6 presents the evaluation of the actors in terms of their attractiveness, likability, intelligence and competence. As intended by design, female 1 was rated 1.1 points less attractive as compared to female 2 (N = 677, t = 7.44, p < 0.001). Similarly, male 1 was rated less attractive as compared to male 2 by 1.2 points on the 11-point scale from 0 to 10 (N = 713, t = 7.90, p < 0.001). Hence, the intended attractiveness variation worked for our female and male actors. Furthermore, the evaluations in terms of actors’ likability, intelligence and competence do not differ statistically significantly between the genders. Fig 7 shows the evaluation in terms of actors’ convincibility. There are differences between the topics with the talk about geoengineering being least convincing. However, and more importantly, the convincibility does not differ between the genders. This is also true for the talk about the pedagogical method.

**Fig 8:**
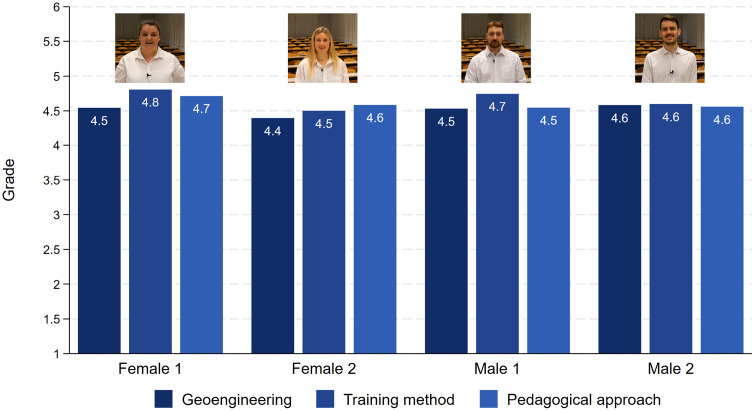
Rating by grades. Note: Female 1: Geoengineering (N = 116), Training methods (N = 93), Pedagogical approach (N = 126). Female 2: Geoengineering (N = 120), Training methods (N = 126), Pedagogical approach (N = 96). Male 1: Geoengineering (N = 111), Training methods (N = 108), Pedagogical approach (N = 134). Male 2: Geoengineering (N = 128), Training methods (N = 113), Pedagogical approach (N = 119).

**Fig 9:**
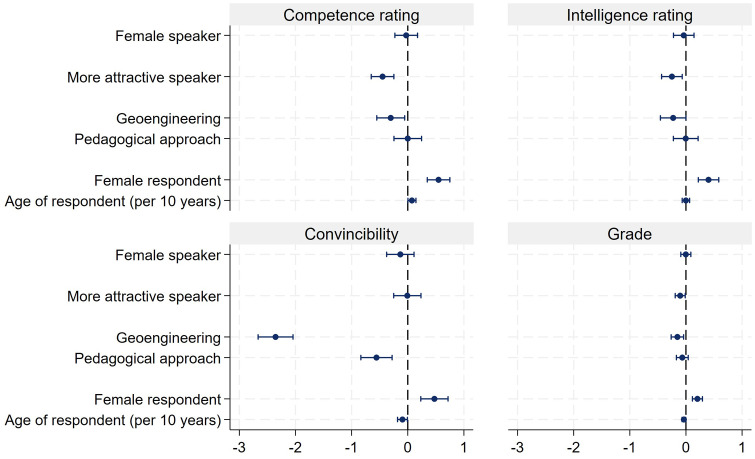
OLS regressions of intelligence, competence, convincibility and grades. Note: N of competence rating = 1,385 (adj. R^2^ = 0.039), N of intelligence rating = 1,385 (adj. R^2^ = 0.019), N of convincibility = 1,385 (adj. R^2^ = 0.165), N of grade = 1,385 (adj. R^2^ = 0.022).

Only within the talk about the training method did the two males receive slightly better evaluations as compared to the two females. All talks receive a sufficient grade between 4.4 and 4.8, and most importantly there are no gender differences (see Fig 8). These descriptive results are also mirrored by multiple OLS regressions in which competence, intelligence, convincibility and grades are the dependent variables (Fig 9, see also S9 Table in S1 File in the supporting information). The most important result is that the female characters never encounter a disadvantage in either of the four evaluations as compared to the male characters. Being more attractive never has an advantage. Quite to the contrary, the more attractive speakers were rated as a little less competent and intelligent. Moreover, female respondents are always a little more generous in their evaluations as compared to male respondents.

**Fig 10:**
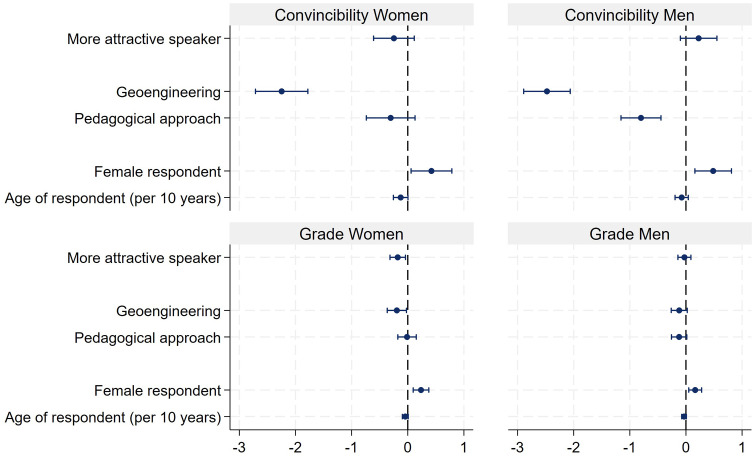
OLS regressions of convincibility and grade separated by gender. Note: N of convincibility for women = 675 (adj. R^2^ = 0.152), N of convincibility for men = 710 (adj. R^2^ = 0.180), N of grade for women = 675 (adj. R^2^ = 0.032), N of grade for men = 710 (adj. R^2^ = 0.013).

To investigate more in detail where the effect of attractiveness stems from, Fig 10 (see also S10 Table in S1 File in the supporting information) presents the results separated by gender. The results show that attractiveness has no effect on the evaluation of the male characters. With respect to the female actors, the result is less clear. Attractiveness has no influence on the competence rating. However, the more attractive female character receives a slightly lower grade.

As with study I we also conducted robustness checks for the regression results of study II. Again, multicollinearity is no problem in the regression analyses (VIF vary between = 1.01 and 1.42). We used robust standard errors for calculating the significance levels of regression coefficients, in order to take care of heteroscedasticity. Furthermore, we checked the age variable for nonlinearity. The results show that there is no nonlinearity with respect to the age effect. Finally, we also checked if statistical outliers bias the estimation results. Also, this test was negative. We also tested if subjects’ prior knowledge of the method did influence any of the reported results. This was not the case. Furthermore, we expanded the regression analyses by controlling for subjects’ traditional gender role attitudes. Again, this did not change any of the reported results (see S11 Table in S1 File in the supporting information). Moreover, separating the sample into two subgroups with traditional and non-traditional gender role attitudes did not find any differential results concerning the (non-present) gender effect.

## 8. Summary and conclusion

In this paper, we present the results of two large-scale online experiments investigating if there are gender differences in performances evaluations. In both studies, respondents watched a 2-minute video in which either a female or male character presented a talk about three different topics. In Study I, respondents (N = 3,157) were students from a large university in Switzerland. We used avatars as presenters and found that the female avatars received even slightly better evaluations than the male avatars. Furthermore, in Study I, we found that the more attractive male avatar received better evaluations than the less attractive male avatar. However, this attractiveness bonus did not apply to the female avatars.

Study I has two obvious limitations: first, it uses a student sample, that is a sample of young and highly educated individuals. Hence, the story Study I entails is that there is no gender bias among young and educated individuals. Second, Study I uses avatars as the presenting characters. Using avatars has the advantage that the presentations are highly standardized between the two female and the two male characters and also between the genders. However, the avatars appear also somewhat artificial, non-human and somewhat like robots. This artificiality might have eliminated any evaluation in terms of gender. Both disadvantages are addressed in the second study which uses a Swiss representative sample (online-panel, N = 1,390) and real actors as presenting characters. Study II replicates the findings of our first study. We do not find any gender differences in terms of the evaluations of the presentations. Furthermore, we also did not find much of an effect of attractiveness. We did not observe any differences for attractiveness for the male actors and mixed results for the female actors. The more attractive female actor received a lower grade but there were no differences with respect to convincibility. Overall, we did not find any gender differences in terms of evaluations in both studies and no consistent effects of attractiveness.

Of course, both studies have limitations. First, both studies were conducted in Switzerland and therefore the results apply only to the Swiss context. It might of course be different for other countries with stronger gender stereotypes. Second, the presentations lasted only for 2 minutes. Most presentations in real life, be it in academics or within market companies last much longer. Hence, the question emerges whether two-minute videos are long enough for ratings in terms of convincibility and quality. We believe it is enough, since we observed strong differences between the topics. Thus, two-minute videos were enough for respondents to give very different evaluations for the geoengineering topic or the other two topics. Third, the type of performances we investigated were oral presentations. Here we find no gender differences. There are many other types of performances such as art, music, or handicraft. Hence, we cannot generalize our findings to all possible types of performances and investigating other types of performances must be left for further research.

Fourth, our measures of performance evaluation like grading the performance or evaluating its convincibility had no consequences for the respondents. Hence, future research would benefit from using incentive compatible measures such as whether respondents would like to receive more information on a topic.

## Supporting information

S1 File Supporting information on gender and the evaluation of performance. (PDF)
