# Supplementary material for: Is the evaluation of performance gender specific? Evidence from two large experimental studies
Source: PLoS One. 2026 Feb 18;21(2):e0336066. doi: 10.1371/journal.pone.0336066 (PMC12915947; doi:10.1371/journal.pone.0336066)

Is the evaluation of performance gender specific? Evidence from two large experimental studies

Supporting information

Table of contents

S1 Table: Text about Geoengineering 1
S2 Table: Text about the pedagogical approach 2
S3 Table: Text about the ski training method 2
S4 Table: Variable description of Study I 3
S5 Table: OLS regression models of Study I 4
S6 Table: OLS regression models of Study I separated by gender 5
S7 Table: Sample comparison of Intervista (Study II), Swiss Household Panel (SHP, W24) and MOSAiCH 2022 6
S8 Table: Variable description of Study II 7
S9 Table: OLS regression models of Study II 8
S10 Table: OLS regression models of Study II separated by gender 9
S11 Table: Further OLS regression models of Study II 10
S12 Table: Variable description of the variables used for further analysis in S11 Table........ 11
S1 Fig: The four presenters (avatars) from synthesia.io 11
S2 Fig: Observed number of observations per experimental condition in Study I 12

S1 Table: Text about Geoengineering

Good afternoon, in the following two minutes I will introduce the Solar Radiation Management approach. The goal of Solar Radiation Management is to intervene in the Earth's geo- and biochemical cycles to regulate the climate. Instead of reducing climate-related emissions, Solar Radiation Management can be used to influence solar radiation in a way that reduces global warming. The average global surface temperature of the Earth is highly dependent on solar radiation. Solar radiation is absorbed by the Earth's atmosphere and surface, causing the Earth to warm. Furthermore, the average temperature of the Earth is affected by thermal radiation. Solar Radiation Management methods start exactly here. Either an attempt is made to reduce the radiation reaching the earth's surface or to reduce the reflectivity of the earth's atmosphere. One possibility is to transport mirrors or reflectors into space. These can then deflect part of the sun's rays so that they no longer hit the earth. The great advantage of this method is that the temperature on Earth could be kept constant or reduced despite an increase in CO_2. This could buy time to reduce the CO_2 emissions that cause climate change. A disadvantage of this method is the enormous mass of mirrors and the resulting logistical effort that would have to be made to transport them into space. Furthermore, the introduction of mirrors into space can lead to irreversible consequences such as a shift in precipitation. In summary, however, it can be stated that solar radiation management predominantly offers advantages to reduce climate change.

S2 Table: Text about the pedagogical approach

Good afternoon, in the following two minutes I will introduce the Marte Meo method. Marte Meo is a therapeutic-pedagogical method in order to improve social interactions. With the support of video recordings, this method aims to promote targeted learning processes in children, parents or pedagogical professionals. In the Marte Meo method, video recordings are made of everyday situations. The therapist then conducts a detailed interaction analysis. This can focus on various skills such as when children can concentrate and maintain eye contact with a conversation partner. Afterwards, small excerpts from the analysis are shown to the participants and discussed with them. The focus is on the moments of interaction that have a particularly positive influence on the course of the conversation. The Marte Meo method has several advantages: In particular, the visualization of the interaction helps to better understand and reflect on a situation. On the one hand, the persons involved receive differentiated feedback about the successful moments of the observed interaction. For example, a teacher can be shown when he or she is particularly successful in attracting a child's attention. On the other hand, concrete suggestions for improvement can be found for problematic moments of interaction. A disadvantage of the method is that the identification and analysis of suitable small sections is very time-consuming. Therefore, the method requires a lot of time and causes high costs. Furthermore, the selection and analysis of suitable interaction sequences is dependent on the therapist's skills and is subject to their subjective interpretation. In summary, however, it can be stated that the Marte Meo method predominantly offers advantages for improving interpersonal communication.

S3 Table: Text about the ski training method

Good afternoon, in the following two minutes I will introduce the idea of Whole-Body Vibration training in alpine ski racing. In ski racing there are constant shocks and vibrations due to uneven slopes, which have to be compensated by the body muscles. This requires a lot of strength, as the stress increases with increasing speed. Consequently, strength training is important for ski racers. However, there is usually little time for long-term strength building of different muscle components. This is where the method of vibratory muscle stimulation comes into play. Here, vibration plates on which the athlete stands generate vibrations similar to those used in skiing. Then the same strength exercises are completed as in conventional training. However, the rapid vibrations activate more muscle fibers at the same time and do so faster. This makes the method more efficient than conventional strength training without additional vibrations. In comparison, with the use of an individualized vibration frequency, increases in maximum strength as well as rapid strength of up to 18% can be achieved. With proper and professional application, vibration training can also significantly improve the damping abilities of athletes. A disadvantage of vibration training is that the physical strain is high, especially in the beginning. In addition, if used incorrectly, there is a risk of injury and whiplash. Some studies also report harmful effects for nerves and increased occurrence of headaches. Because of this, choosing the right frequency and the number and duration of training intervals are crucial. In summary, however, it can be stated that the vibration method predominantly offers advantages to complement training in the high-performance area of ski racing.

S4 Table: Variable description of Study I

Variable	Description	Obs.	Min.	Max.	Mean	SD
Experimental conditions (12)						
Gender of presenter	0 = male<br>1 = female	3,157	0	1	48.8%<br>51.2%	
Attractiveness of presenter	0 = less attractive<br>1 = more attractive	3,157	0	1	52.3%<br>47.7%	
Topic of the talk	0 = Geoengineering<br>1 = Training method<br>2 = Pedagogical approach	3,157	0	2	34.2%<br>33.6%<br>32.2%	
Evaluation of the presenters						
Attractive	0 = not at all, 10 = very much	3,025	0	10	4.7	2.50
Likable	0 = not at all, 10 = very much	3,022	0	10	4.7	2.35
Intelligent	0 = not at all, 10 = very much	3,021	0	10	5.9	2.12
Competent	0 = not at all, 10 = very much	3,024	0	10	6.2	2.23
Evaluation of the talk						
Grade	1 = very bad, 1.5, 2, 2.5, 3, 3.5, 4. 4.5, 5, 5.5, 6 = excellent	3,027	1	6	4.4	0.97
Convincibility	0 = not at all convincing, 10 = very convincing	3,028	0	10	5.2	2.15
Characteristics of respondents						
Gender	0 = male<br>1 = female<br>2 = diverse	3,026	0	2	33.5%<br>65.5%<br>1%	
Age	In years, calculated with birth year	3,026	18	61	24.5	4.38

Obs. = number of observations, min. = minimum, max. = maximum, SD = standard deviation.

S5 Table: OLS regression models of Study I

	(1) Competence rating	(2) Intelligence rating	(3) Convincibility	(4) Grade
Female speaker	0.174*	0.218**	0.097	0.101**
	(0.082)	(0.077)	(0.075)	(0.035)
More attractive speaker	-0.029	-0.066	-0.000	0.205***
	(0.083)	(0.077)	(0.075)	(0.035)
Topic				
Geoengineering	-0.307**	0.005	-1.448***	-0.113**
	(0.102)	(0.095)	(0.096)	(0.042)
Pedagogical approach	-0.114	0.113	-0.438***	-0.138***
	(0.101)	(0.091)	(0.086)	(0.042)
Female respondent	0.392***	0.268**	0.187*	0.088*
	(0.090)	(0.083)	(0.083)	(0.038)
Age of respondent (per 10 years)	-0.324**	-0.337***	-0.189*	0.015
	(0.106)	(0.102)	(0.095)	(0.043)
Constant	6.820***	6.467***	6.145***	4.196***
	(0.285)	(0.267)	(0.258)	(0.117)
Observations	2987	2984	2992	2991
Adjusted R^2	0.014	0.011	0.084	0.018
F	7.572	5.487	39.816	9.889
P-value	0.000	0.000	0.000	0.000

The table displays unstandardized regression coefficients. Standard errors are in parentheses.
F[1] (6, 2980), F[2] (6, 2977), F[3] (6, 2985), F[4] (6, 2984).
** p < 0.05, ** p < 0.01, *** p < 0.001.*

S6 Table: OLS regression models of Study I separated by gender

	(1)<br>Convincibility<br>Women	(2)<br>Convincibility<br>Men	(3)<br>Grade<br>Women	(4)<br>Grade<br>Men
More attractive speaker	-0.268**	0.286*	0.011	0.413***
	(0.101)	(0.111)	(0.046)	(0.051)
Topic				
Geoengineering	-1.441***	-1.450***	-0.135*	-0.082
	(0.130)	(0.142)	(0.057)	(0.061)
Pedagogical approach	-0.382***	-0.507***	-0.100	-0.187**
	(0.115)	(0.127)	(0.055)	(0.063)
Female respondent	0.188	0.191	0.044	0.143*
	(0.109)	(0.125)	(0.050)	(0.058)
Age of respondent (per 10 years)	-0.245<br>(0.130)	-0.126<br>(0.140)	0.032<br>(0.050)	-0.004<br>(0.069)
Constant	6.488***	5.878***	4.375***	4.112***
	(0.343)	(0.376)	(0.140)	(0.183)
Observations	1541	1451	1540	1451
Adjusted R^2	0.093	0.081	0.001	0.049
F	28.345	23.066	1.518	15.900
P-value	0.000	0.000	0.181	0.000

The table displays unstandardized regression coefficients. Standard errors are in parentheses.
F[1, 2, 4] (5.1535), F[3] (5, 1534).
** p < 0.05, ** p < 0.01, *** p < 0.001.*

S7 Table: Sample comparison of Intervista (Study II), Swiss Household Panel (SHP, W24) and MOSAiCH 2022

	Intervista	SHP W24[1]	MOSAiCH 2022[2]
Gender (percent of women)	49.51	51.47	51.58
Age (mean)	42.56 (13.54)	43.98*** (14.39)	43.98** (13.13)
Education (percent of tertiary education)	48.11	45.06**	50.58
Employment (percent of employed)	77.52	87.49***	84.32***
Percent of Non-Swiss citizens	7.4	8.24	16.55***
Marriage (percent of individuals married)	41.13	52.34***	52.68***
Number of household members (mean)	2.65 (1.28)	2.58* (1.26)	2.77** (1.27)
Equivalized household monthly income (mean)	5,900 (2,417)	6,749*** (2,386)	
Left-right (0 = left, 10 = right) (mean)	4.78 (2.18)	4.71 (2.12)	
General trust (0 = can't be too careful, 10 = can trust most people) (mean)	5.53 (2.14)	6.45*** (2.05)	
Life satisfaction (0 = not at all satisfied, 10 = completely satisfied) (mean)	7.55 (1.70)	8.0*** (1.32)	
Equal opportunities for men and women in CH (1 = strongly agree, 5 = strongly disagree) (mean)	3.12 (1.1)		3.44*** (1.06)
Child suffers from mother's job (1 = strongly agree, 5 = strongly disagree) (mean)	3.56 (1.17)		3.44** (1.18)
Men's job is earning money, women's job is household (1 = strongly agree, 5 = strongly disagree) (mean)	4.25 (0.95)		4.13*** (1.05)

Standard deviations in paratheses. T-tests for sample differences in means, and z-tests for sample differences of proportions.
*Significant differences to Intervista sample: * $p < 0.05$, ** $p < 0.01$, *** $p < 0.001$.*

[1] SHP Group. (2024). Living in Switzerland Waves 1-24 (including a long file) + Covid 19 data + Beta version wave 25 (Version 8.0) [Data set]. FORS. https://doi.org/10.48573/n4ns-5830

[2] Michèle Ernst Stähli, Marlène Sapin, Alexandre Pollien, Michael Ochsner, Karin Nisple (2023). MOSAiCH 2022. Measurement and Observation of Social Attitudes in Switzerland. Study on Family and Changing Gender Roles and related topics (1.0.0) [Dataset]. FORS - Swiss Centre of Expertise in the Social Sciences. Distributed by FORS, Lausanne. https://doi.org/10.48573/6ezs-eg91

S8 Table: Variable description of Study II

Variable	Description	Obs.	Min.	Max.	Mean	SD
Experimental conditions (12)						
Gender of presenter	0 = male<br>1 = female	1,390	0	1	51.3%<br>48.7%	
Attractiveness of presenter	0 = less attractive<br>1 = more attractive	1,390	0	1	49.5%<br>50.5%	
Topic of the talk	0 = Geoengineering<br>1 = Training method<br>2 = Pedagogical approach	1,390	0	2	34.2%<br>31.7%<br>34.2%	
Evaluation of the presenters						
Attractive	0 = not at all, 10 = very much	1,390	0	10	5.7	2.14
Likable	0 = not at all, 10 = very much	1,390	0	10	7.0	1.91
Intelligent	0 = not at all, 10 = very much	1,390	0	10	7.1	1.74
Competent	0 = not at all, 10 = very much	1,390	0	10	7.1	1.95
Evaluation of the talk						
Grade	1 = very bad, 1.5, 2, 2.5, 3, 3.5, 4. 4.5, 5, 5.5, 6 = excellent	1,390	1	6	4.6	0.85
Convincibility	0 = not at all convincing, 10 = very convincing	1,390	0	10	5.6	2.52
Characteristics of respondents						
Gender	0 = male<br>1 = female<br>2 = diverse	1,390	0	2	51.4%<br>48.2%<br>0.4%	
Age	In years, calculated with birth year	1,390	18	65	42.4	13.62

Obs. = number of observations, min. = minimum, max. = maximum, SD = standard deviation. The descriptive statistics refer to the respondents who viewed the videos with the real actors.

S9 Table: OLS regression models of Study II

	(1) Competence rating	(2) Intelligence rating	(3) Convincibility	(4) Grade
Female speaker	-0.028	-0.039	-0.132	-0.002
	(0.103)	(0.093)	(0.124)	(0.046)
More attractive speaker	-0.449^{***}	-0.250^{**}	-0.007	-0.102^{*}
	(0.103)	(0.094)	(0.124)	(0.045)
Topic				
Geoengineering	-0.303^{*}	-0.228^{*}	-2.355^{***}	-0.152^{**}
	(0.127)	(0.116)	(0.158)	(0.056)
Pedagogical approach	0.000	-0.004	-0.557^{***}	-0.065
	(0.126)	(0.112)	(0.142)	(0.054)
Female respondent	0.549^{***}	0.402^{***}	0.475^{***}	0.205^{***}
	(0.103)	(0.093)	(0.124)	(0.045)
Age of respondent (per 10 years)	0.075^{*}	-0.001	-0.094^{*}	-0.037^{*}
	(0.036)	(0.034)	(0.045)	(0.017)
Constant	6.909^{***}	7.091^{***}	6.862^{***}	4.770^{***}
	(0.196)	(0.181)	(0.232)	(0.087)
Observations	1385	1385	1385	1385
Adjusted R^2	0.039	0.019	0.165	0.022
F (6, 1378)	10.987	6.015	43.052	6.452
P-value	0.000	0.000	0.000	0.000

The table displays unstandardized regression coefficients. Standard errors are in parentheses.
* $p < 0.05$, ** $p < 0.01$, *** $p < 0.001$.

S10 Table: OLS regression models of Study II separated by gender

	(1)<br>Convincibility<br>Women	(2)<br>Convincibility<br>Men	(3)<br>Grade<br>Women	(4)<br>Grade<br>Men
More attractive speaker	-0.246	0.226	-0.178*	-0.027
	(0.185)	(0.166)	(0.070)	(0.059)
Topic				
Geoengineering	-2.246***	-2.476***	-0.194*	-0.119
	(0.238)	(0.211)	(0.087)	(0.073)
Pedagogical approach	-0.304	-0.801***	-0.013	-0.121
	(0.221)	(0.181)	(0.084)	(0.070)
Female respondent	0.422*	0.485**	0.236***	0.165**
	(0.185)	(0.166)	(0.071)	(0.058)
Age of respondent (per 10 years)	-0.126	-0.076	-0.044	-0.034
	(0.066)	(0.060)	(0.027)	(0.021)
Constant	6.893***	6.792***	4.818***	4.750***
	(0.344)	(0.301)	(0.132)	(0.109)
Observations	675	710	675	710
Adjusted R^2	0.152	0.180	0.032	0.013
F	23.344	31.150	5.269	3.103
P-value	0.000	0.000	0.000	0.009

The table displays unstandardized regression coefficients. Standard errors are in parentheses.
F[1, 3] (5, 669), F[2, 4] (5, 704).
* $p < 0.05$, ** $p < 0.01$, *** $p < 0.001$

S11 Table: Further OLS regression models of Study II

	(1) Competence rating	(2) Intelligence rating	(3) Convincibility	(4) Grade
Female speaker	-0.021	-0.036	-0.135	-0.002
	(0.103)	(0.093)	(0.125)	(0.046)
More attractive speaker	-0.455***	-0.251**	0.008	-0.103*
	(0.103)	(0.094)	(0.124)	(0.046)
Geoengineering	-0.301*	-0.227	-2.340***	-0.151**
	(0.127)	(0.116)	(0.158)	(0.057)
Pedagogical approach	-0.008	0.004	-0.491***	-0.060
	(0.128)	(0.115)	(0.145)	(0.056)
Female respondent	0.565***	0.411***	0.471***	0.203***
	(0.105)	(0.095)	(0.125)	(0.046)
Age of respondent (per 10 years)	-0.301	0.084	-0.132	-0.111
	(0.261)	(0.244)	(0.329)	(0.121)
Age of respondent (per 10 years)^2	0.044	-0.011	0.005	0.009
	(0.032)	(0.029)	(0.039)	(0.015)
Tertiary education	0.043	0.106	-0.191	0.013
	(0.113)	(0.101)	(0.135)	(0.049)
Prior knowledge of method	-0.035	0.027	0.299*	0.014
	(0.106)	(0.098)	(0.133)	(0.048)
Traditional gender attitudes	0.080	-0.026	0.029	-0.019
	(0.109)	(0.100)	(0.135)	(0.050)
Constant	7.578***	6.884***	6.854***	4.903***
	(0.476)	(0.461)	(0.629)	(0.223)
Observations	1385	1385	1385	1385
Adjusted R^2	0.037	0.017	0.167	0.020
F (10, 1374)	6.811	3.825	27.335	3.921
P-value	0.000	0.000	0.000	0.000

The table displays unstandardized regression coefficients. Standard errors are in parentheses.
** p < 0.05, ** p < 0.01, *** p < 0.001.*

S12 Table: Variable description of the variables used for further analysis in S11 Table

Variable	Description	Obs.	Min.	Max.	Mean
Tertiary education	0 = No tertiary education<br>1 = Tertiary education<br>*Tertiary education includes the following degrees: university or ETH/EPF, university of applied science (FH/HES), university of teacher education (PH/HEP), (Advanced) Federal Diploma of Higher Education*	1,390	0	1	47.2%
Prior knowledge of the method	0 = No prior knowledge [0]<br>1 = Prior knowledge [1-10]<br>*Original sale from 0 “No prior knowledge” to 10 “Very good prior knowledge”*	1,390	0	1	36.8%
Egalitarian gender role norms	Additive index from two items “Child suffers from a mother’s job” an “Men’s job is earning money, women’s job is household” (1 = strongly agree, 5 = strongly disagree)	1,390	2	10	7.81
Traditional gender role norms	0 = Egalitarian gender role attitudes<br>1 = Traditional gender role attitudes<br>*Based on the scale for egalitarian gender role norms traditional views (1) include the values from 2 to 7 and egalitarian views (0) the values from 8 to 10*	1,390	0	1	38.4%

S1 Fig: The four presenters (avatars) from synthesia.io

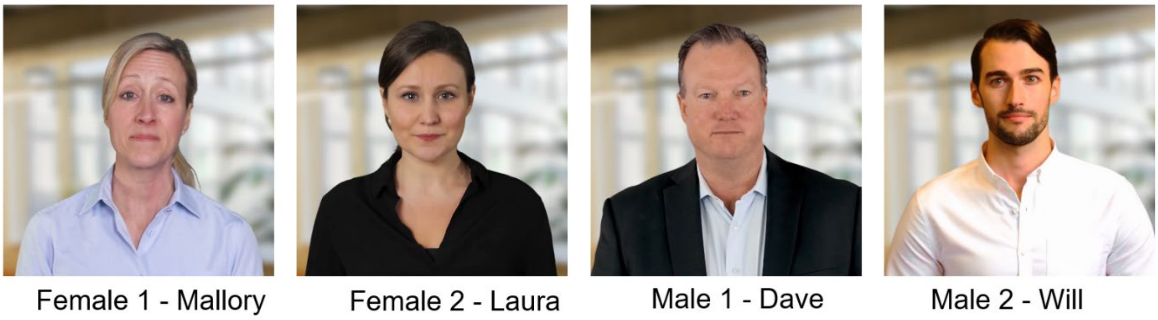

Note: Laura was rated as being more attractive than Mallory, and Will was rated as being more attractive than Dave.

S2 Fig: Observed number of observations per experimental condition in Study I

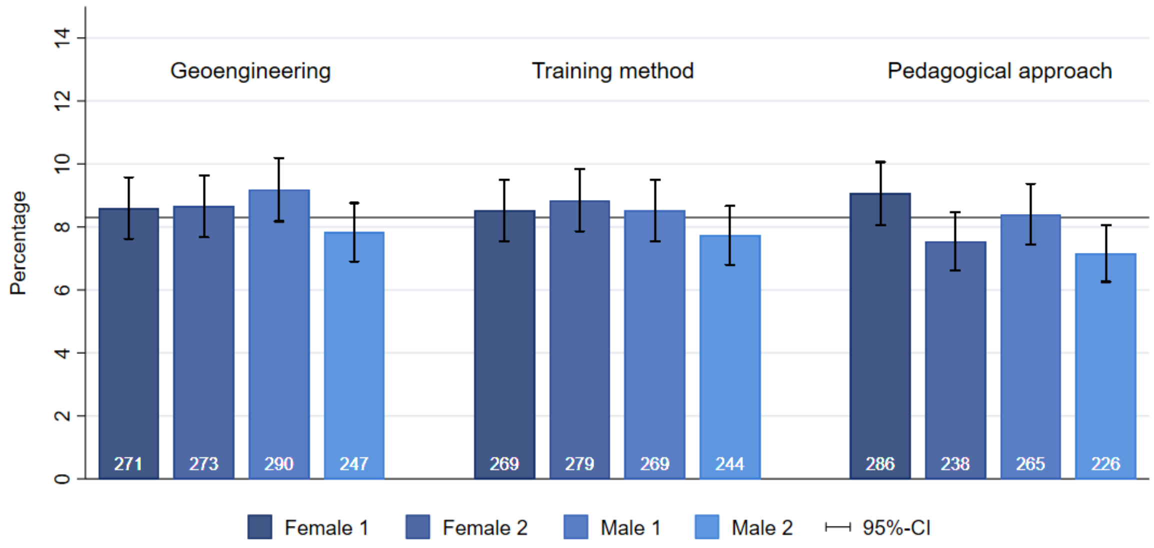

Supplement: S1 File — (PDF) [file pone.0336066.s001.pdf]
